# Using machine learning to gain insights into chronic disease multimorbidity: trends and patterns in British Columbia, Canada

**DOI:** 10.23889/ijpds.v9i5.2744

**Published:** 2024-09-18

**Authors:** Jennifer Ferris, Alexandra Choi, Brandon Wagar, Jonathan Simkin, Ryan Woods, Ognjenka Djurdjev, Hind Sbihi, Kari Harder, Kate Smolina

**Affiliations:** 1 BC Centre for Disease Control, Provincial Health Services Authority; 2 Gerontology Research Centre, Simon Fraser University; 3 Vancouver Coastal Health Authority; 4 Health Sector Information, Analysis and Reporting (HSIAR), BC Ministry of Health; 5 Provincial Health Services Authority; 6 Northern Health Authority

## Objective

The goal of this project is to explore novel ways to assess chronic disease multimorbidity (co-occurrence of two or more conditions) trends and patterns in the population of British Columbia (BC), Canada.

## Approach

This study included linked data from the BC population (~5M individuals) from 2001/02 to 2019/20.  We analysed 25 chronic conditions, including 7 primary cancer subtypes. We report multimorbidity (MM) incidence, prevalence, and most common disease combinations. Further we explore temporal MM disease patterns using directed network analyses and extracted data-driven disease clusters with an unsupervised machine learning algorithm.

## Results

The age-standardized incidence of MM stayed relatively stable over the study period for males and decreased for females, while prevalence increased to approximately 1 in 4 individuals in BC in 2019/20 (from 19% to 27% of females, from 15% to 22% of males). Disease networks and clusters varied significantly by sex and age group, this presentation will highlight select disease network and cluster findings and discuss their implications for chronic disease surveillance.

## Conclusions

The prevalence of multimorbidity continues to rise in BC. Using advanced analytics to understand disease co-occurrence patterns provides new insights above and beyond traditional epidemiological metrics to support health system planning and prevention efforts.

## Implications

Chronic disease surveillance and research have historically operated with a single disease focus, which is not patient-centered and does not adequately account for the reality of multimorbidity for many people. This project is laying the foundation for enhanced chronic disease surveillance and monitoring in BC.

